# Nelfinavir Impairs Glycosylation of Herpes Simplex Virus 1 Envelope Proteins and Blocks Virus Maturation

**DOI:** 10.1155/2015/687162

**Published:** 2015-01-29

**Authors:** Soren Gantt, Eliora Gachelet, Jacquelyn Carlsson, Serge Barcy, Corey Casper, Michael Lagunoff

**Affiliations:** ^1^Seattle Children's Research Institute, University of Washington, Seattle, WA 98101, USA; ^2^Department of Pediatrics, University of Washington, Seattle, WA 98105, USA; ^3^Department of Global Health, University of Washington, Seattle, WA 98195, USA; ^4^Department of Microbiology, University of Washington, Seattle, WA 98195, USA; ^5^Department of Medicine, University of Washington, Seattle, WA 98195, USA; ^6^Department of Epidemiology, University of Washington, Seattle, WA 98195, USA; ^7^Fred Hutchinson Cancer Research Center, Seattle, WA 98109, USA

## Abstract

Nelfinavir (NFV) is an HIV-1 aspartyl protease inhibitor that has numerous effects on human cells, which impart attractive antitumor properties. NFV has also been shown to have *in vitro* inhibitory activity against human herpesviruses (HHVs). Given the apparent absence of an aspartyl protease encoded by HHVs, we investigated the mechanism of action of NFV herpes simplex virus type 1 (HSV-1) in cultured cells. Selection of HSV-1 resistance to NFV was not achieved despite multiple passages under drug pressure. NFV did not significantly affect the level of expression of late HSV-1 gene products. Normal numbers of viral particles appeared to be produced in NFV-treated cells by electron microscopy but remain within the cytoplasm more often than controls. NFV did not inhibit the activity of the HSV-1 serine protease nor could its antiviral activity be attributed to inhibition of Akt phosphorylation. NFV was found to decrease glycosylation of viral glycoproteins B and C and resulted in aberrant subcellular localization, consistent with induction of endoplasmic reticulum stress and the unfolded protein response by NFV. These results demonstrate that NFV causes alterations in HSV-1 glycoprotein maturation and egress and likely acts on one or more host cell functions that are important for HHV replication.

## 1. Introduction

Human herpesvirus (HHV) infections are ubiquitous and are responsible for substantial morbidity and mortality worldwide, particularly among people infected with human immunodeficiency virus (HIV). Herpes simplex virus (HSV) and cytomegalovirus (CMV) infections can be recurrent and difficult to treat in HIV coinfected individuals [1]. Moreover, genital HSV infection has been associated with greater risks of HIV acquisition, transmission, and progression of disease [2]. HHV-8 and Epstein-Barr virus infections cause the most common AIDS-defining malignancies, Kaposi sarcoma and non-Hodgkin lymphoma, respectively [3]. Although greatly reduced by effective antiretroviral therapy (ART), complications of HHV infections remain among the most common medical problems in people infected with HIV worldwide [3–7].

Currently available antiviral drugs to treat or prevent complications of HHV infections all directly or indirectly target the viral polymerase [8]. Each of these drugs has one or more important limitations, including selection of drug-resistant viral mutants, significant toxicities, and/or poor bioavailability requiring intravenous administration. For example, treatment of acyclovir-resistant HSV or ganciclovir-resistant CMV infections requires the use of intravenous foscarnet or cidofovir, both of which are associated with nephrotoxicity. As such, new agents that are effective for HHV infections are needed that are safe, orally bioavailable and have a high barrier to resistance.

Nelfinavir (NFV) is a first-generation HIV aspartyl protease inhibitor recently found to block production of multiple HHVs [9]. Furthermore, because it also has potent antitumor and antiangiogenic properties, clinical trials are ongoing to evaluate NFV for the treatment of several cancers [10–15]. The mechanisms by which NFV acts on tumor cells are multifactorial and include inhibition of cellular proteases, Akt activation, and NF*κ*-B signaling, as well as induction of the endoplasmic reticulum (ER) stress, the unfolded protein response (UPR), and autophagy [11, 16, 17].

In contrast to the aspartyl protease required for HIV maturation, HHVs utilize a serine protease, which for HSV-1 is the gene product of UL26 open reading frame (ORF) [18]. As such, we hypothesized that NFV does not inhibit HHV replication by acting on the viral protease. Furthermore, we speculated that NFV acting on a nonprotease viral target would be improbable and that its antiviral activity is more likely due to one or more of its effects on host cells, which could impair efficient viral replication. We therefore investigated mechanism of action of NFV on HSV-1, by attempting to identify host cell or viral targets of the drug* in vitro*.

## 2. Methods

### 2.1. Cells and Virus

Human fibroblasts (HF) were cultivated in Dulbecco's modified Eagle's medium (DMEM; Gibco) containing 10% fetal bovine serum (FBS) and 100 units per mL penicillin G and 100 *μ*g per mL streptomycin (Pen-Strep) and maintained at 37°C in a humidified 5% CO_2_ atmosphere, as previously described [9]. HEK293-T cells were maintained in DMEM supplemented with 10% FBS and Pen-Strep. HSV-1 (strain F) was a gift from Keith Jerome (Fred Hutchinson Cancer Research Center).

### 2.2. Drugs

NFV and indinavir (IDV) were obtained through the AIDS Research and Reference Reagent Program, Division of AIDS, NIAID, NIH. NFV was solubilized in dimethyl sulfoxide (DMSO). IDV and acyclovir (Sigma-Aldrich) were solubilized in water.

### 2.3. Antibodies

The primary antibodies used were as follows: mouse monoclonal anti-HSV gB (Virusys), mouse monoclonal anti-HSV1 gC clone 3G9 (Abcam), rabbit polyclonal anti-LC3B (Cell Signaling Technology), mouse monoclonal anti-*β*-actin, anti-FLAG M2 monoclonal, and anti-HA monoclonal antibody, clone HA-7 (Sigma-Aldrich). For Western blotting, the secondary antibodies used were peroxidase-conjugated AffiniPure F(ab′)2 fragment of goat anti-mouse IgG(H + L) or peroxidase-conjugated AffiniPure F(ab′)2 fragment of goat anti-rabbit IgG(H + L) (Jackson ImmunoResearch). Secondary antibodies for immunofluorescence were AlexaFluor 594 F(ab′)2 fragment of goat anti-mouse IgG(H + L) or AlexaFluor 488 F(ab′)2 fragment of goat anti-rabbit IgG(H + L) (Life Technologies).

### 2.4. Lectins and Eastern Blotting

Biotinylated lectins used in the Eastern blots (Vector Laboratories, Inc.) were as follows: peanut agglutinin (PNA),* Ricinus communis* agglutinin I (RCA I), wheat germ agglutinin (WGA), and concanavalin A (ConA). Total cellular proteins (0.75–1.0 *µ*g loaded per lane) were separated by SDS-PAGE and transferred to a PVDF membrane. The membranes were blocked with 5% BSA in Tris-buffered saline with 0.3% Tween-20 (TBS-T), incubated with 1-2 *µ*g lectin/mL in blocking buffer for 1-2 hours, washed three times in TBS-T, and then incubated 1 hour with horseradish-peroxidase-conjugated avidin D (Vector Labs) at 1-2 *µ*g/mL in TBS-T. The blots were washed three times in TBS-T following the avidin incubation. Detection was performed using an enhanced chemiluminescence method (Pierce ECL Plus).

### 2.5. Selection of Resistance

HF were infected with HSV-1 at an MOI of 0.1 for 1 hour at 37°C and then incubated at 37°C with either 5 *μ*M NFV, 1 *μ*M ACV, or no drug for 3–5 days until most cells were lysed. Four passages were made for each group. Virus was harvested with 3 freeze-thaw cycles followed by the addition of nonfat milk. Virus titers were determined by plaque assay previously described [9].

### 2.6. HSV-1 Protease Activity Assay

The effect of NFV on HSV-1 maturational protease activity was assayed by transfecting HEK293-T cells with vectors expressing the protease (VP24; N-terminal 247 amino acids of the UL26 ORF gene product), as well as two substrates: the HSV-1 capsid scaffold protein (amino acids 307–635 of the UL26 ORF gene product; full-length product of UL26.5; ICP35) and a catalytically inactive mutant (S129A) of the full-length protease-scaffold protein transcript (amino acids 1–635 of the UL26 ORF gene product) [18]. The protease coding sequence contained an N-terminal HA tag and each substrate contained an N-terminal FLAG tag; all vectors were synthesized by Blue Heron Biotech, LLC. Each substrate was expressed alone or in combination with the protease, in the presence of NFV or a vehicle control. Expression was under the control of the CMV6 promoter. The plasmid pEGFP-N2, which expresses a GFP variant from the CMV immediate-early promoter, was used to monitor transfection efficiencies. Transfection was performed using Mirus* Trans*IT-293 reagent (MIR 2700) according to the manufacturer's instructions. NFV (10 *µ*M final concentration) or DMSO (0.1% final concentration) was added to the cells concurrently with the DNA and transfection reagent. Cultures were incubated 24 hours with no change of medium, at which time the cells were harvested on ice by scraping into RIPA buffer containing protease inhibitors (Roche Complete Mini EDTA-Free Protease Inhibitor Cocktail tablets). Protein concentrations were determined by BCA assay (Pierce). Extracted proteins (10 *µ*g/lane) were separated by SDS-PAGE and transferred to PVDF membranes by tank transfer overnight. The blots were blocked with 5% nonfat dry milk and probed with anti-FLAG M2 mouse monoclonal antibody (Sigma), recognizing the tag on the scaffold protein, anti-HA monoclonal antibody, clone HA-7 (Sigma), and recognizing the tag on VP24.

### 2.7. Transmission Electron Microscopy

Human foreskin fibroblasts (HFFs) were mock-infected or infected with HSV-1 (strain F) at an MOI of 10 or 50. After 1 hour, the inoculum was replaced with medium containing 0.1% DMSO or 10 *µ*M NFV. At 16 or 20 hpi the medium was replaced with a 1 : 1 mixture of DMEM and 1/2 Karnovsky's fixative [19] and the culture was returned to the incubator for 10 minutes. The mixture was replaced by 1/2 Karnovsky's, and the cells were incubated a further 30–60 minutes at room temperature. The cells were scraped, transferred to a microcentrifuge tube, and pelleted at 200 ×g. The pellet was resuspended in 1 mL of fixative and then dehydrated, embedded, sectioned, and affixed to grids according to standard methods. The grids were examined on a JEOL 1230 or a JEOL JEM 1400 transmission electron microscope at the Electron Microscopy Lab at the Fred Hutchinson Cancer Research Center.

### 2.8. Immunofluorescence Microscopy

HFFs were seeded in 4-well chamber slides to a confluence of approximately 70%. They were mock-infected or infected with HSV-1 at an MOI of 3. After 1 h, the inoculum was replaced by medium with 0.1% DMSO or 10 *µ*M NFV, and the cultures were returned to the incubator. Sixteen hours after infection, the cells were fixed in 4% paraformaldehyde in phosphate-buffered saline. Cells were then permeabilized with 0.2% Tween-20. Endogenous peroxidase activity was inhibited with 3% H_2_O_2_. The cells were then incubated with 10% nonfat dry milk containing 1% normal goat serum (blotto/NGS; Jackson) to block nonspecific binding. Primary antibody binding was revealed with anti-mouse IgG-HRP (Jackson) followed by a 10-minute TSA amplification with TSA-594 (Life Technologies). The nuclei were stained with TO-PRO 3. Slides were mounted after addition of SlowFade (Life Technologies) and analyzed by confocal microscopy. Confocal images were generated on an LSM 5 Pascal system (Zeiss).

## 3. Results

### 3.1. NFV Inhibits HSV-1 Replication by a Mechanism Distinct from That of Acyclovir

**Figure 1:**
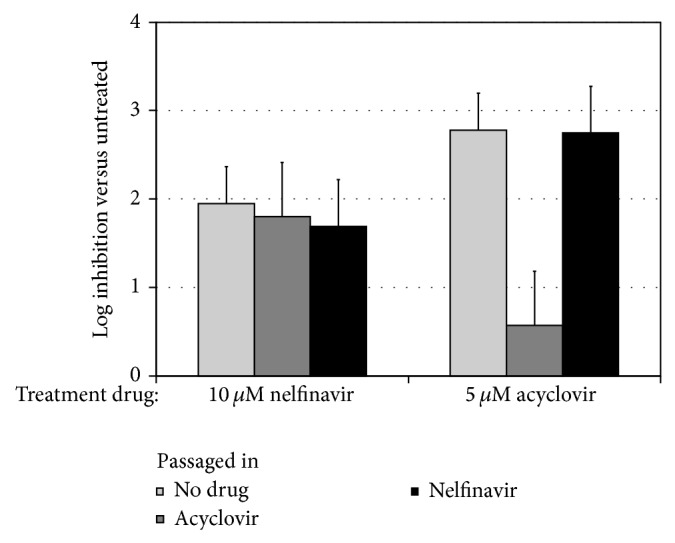
NFV inhibits production of infectious HSV-1 through a mechanism distinct from that of acyclovir and does not readily select for antiviral resistance* in vitro*. HSV-1 was passaged four times in HF in the presence of either 5 *μ*M NFV or 1 *μ*M acyclovir, the approximate IC_50_ of each drug. Virus passaged in acyclovir showed increased resistance to inhibition by acyclovir but was inhibited by NFV similarly to the control HSV-1 isolate. In contrast, passage in the presence of NFV did not result in a significant change in susceptibility to either drug. Shown are the results from three separate experiments.

We passaged HSV-1 in the presence of NFV to determine if resistant mutants could be selected in an attempt to elucidate the mechanism of action of NFV on HHV production. As expected, [20] multistep passage under drug pressure was readily selected for high-level resistance to acyclovir (Figure 1). In contrast, no significant change in susceptibility to NFV was observed despite parallel passaging in the presence of NFV. Of note, the inhibitory activity of NFV was not different between acyclovir-resistant and wild type isolates, further suggesting a distinct antiviral mechanism.

### 3.2. NFV Does Not Inhibit HSV-1 Protease Activity

**Figure 2:**
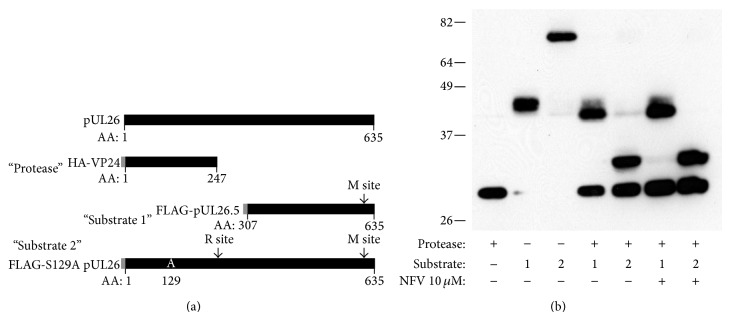
NFV does not inhibit activity of the HSV-1 protease. The coding sequence of the full-length protein product of UL26 ORF (pUL26) and the expression proteins employed are diagramed in panel (a). Transfection vectors were constructed to express the VP24 protease with an N-terminal HA tag (“protease”), the pUL26.5 with an N-terminal FLAG tag (Substrate 1), or the S129A pUL26 mutant inactive protease-scaffold protein with an N-terminal FLAG tag (Substrate 2). Tags are depicted in grey at the left end of each construct. The release (R) and maturation (M) cleavage sites are shown. After transfection into HEK293-T cells, construct expression and substrate cleavage were evaluated by Western blot using anti-HA and FLAG antibodies. Each protein expressed alone was of the expected size (28.8, 37.9, and 70.6 kD, resp., including tag). Similarly, coexpression of protease with each of the 2 substrates resulted in the N-terminal cleavage products of the expected sizes. The generation of substrate cleavage products was not affected by the addition of NFV at concentrations that inhibit HSV-1 replication* in vitro*.

To determine if NFV, an aspartyl protease inhibitor, could act on the essential HSV-1 UL26 serine protease, we tested whether NFV could inhibit the activity of the HSV-1 protease on its scaffold protein substrates using a cotransfection assay. At NFV concentrations that potently block production of infectious HSV-1, there was no effect on the activity of the HSV-1 protease expressed in HEK293T cells (Figure 2).

### 3.3. Inhibition of HSV-1 Replication by NFV Cannot Be Attributed to a Decrease in Akt Activation

**Figure 3:**
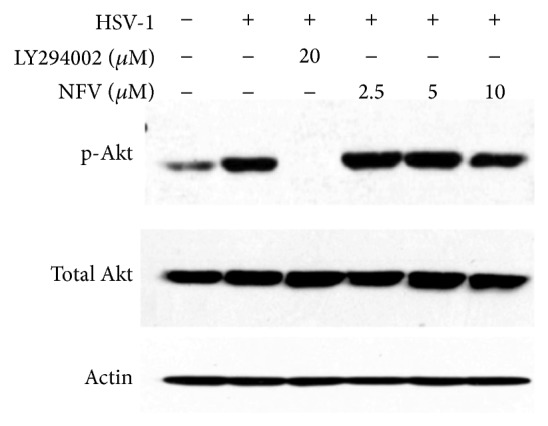
Decreased Akt activation does not account for the ability of NFV to inhibit HSV-1 viral replication. HSV-1 infection of Vero cells resulted in an increase in levels of phosphorylated (p)-Akt at 2 hours compared to uninfected cells. Although the Akt inhibitor LY294002 could completely suppress Akt phosphorylation in HSV-1 infected cells, NFV did not substantially reduce the levels of p-Akt even at drugs concentrations that potently block virus production.

One of the prominent effects of NFV on human cells that has been described is inhibition of the Akt signaling pathway by reducing the phosphorylation of Akt by phosphatidylinositol-3 kinase [21–23]. HSV-1 infection results in an increase in the level of Akt phosphorylation (Figure 3), as has been previously described [24]. The Akt inhibitor LY294002 completely suppressed Akt phosphorylation in HSV-1 infected cells, but NFV did not reduce the levels of phosphorylated Akt even at drug concentrations that potently block virus production (Figure 3). Furthermore, LY294002 treatment of HSV-1 infected HF cells did not reduce the production of infectious virus by plaque assay (not shown), consistent with published data [24]. Therefore, it is unlikely that the documented inhibition of NFV on AKT activation plays a role in the drug's inhibition of HSV-1.

### 3.4. Ultrastructure of NFV-Treated Cells Infected with HSV-1

**Figure 4:**
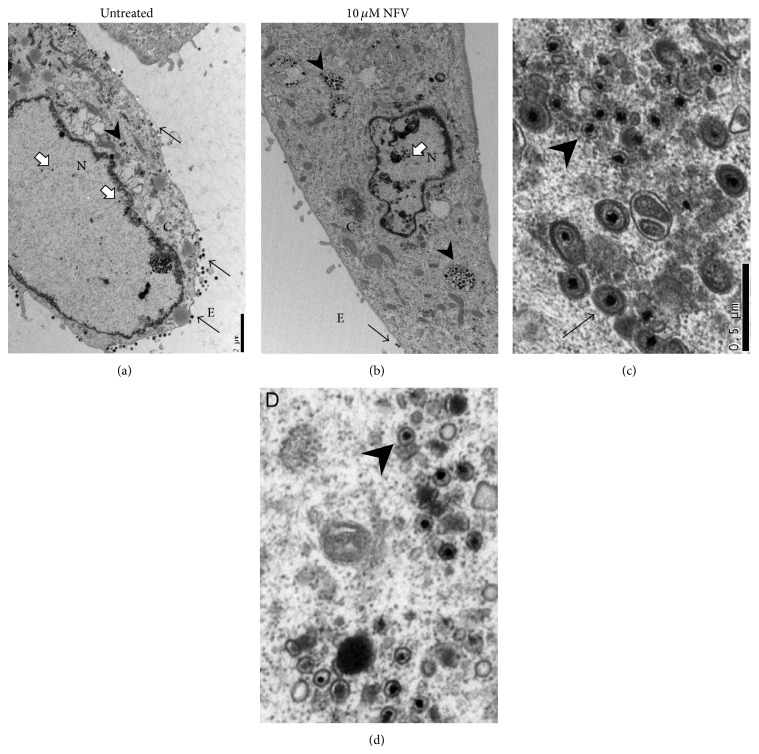
NFV treatment impairs HSV-1 maturation and egress. Shown are representative transmission election micrographs, performed 20 hours after HSV-1 infection of HF that were either untreated (panels (a) and (c)) or treated with 10 *μ*M NFV (panels (b) and (d)). Low power micrographs are shown in panels (a) and (b); bar = 2 *μ*m. White arrows indicate HSV-1 capsids in the nucleus (N), black arrowheads indicate capsids in the cytoplasm (C), and black arrows indicate extracellular (E) virions. Similar numbers of capsids were observed in the nucleus of untreated and NFV-treated cells (approximately 39 and 44, resp.). However, untreated cells were observed to have fewer capsids in the cytoplasm (~14 versus 79) and substantially more extracellular virus particles (~51 versus 3). Cytoplasmic virus particles are shown in panels (c) and (d); bar = 0.5 *μ*m. Enveloped virus particles (indicated in panel (c) by a black arrow) were commonly observed in untreated cells but rarely in NFV-treated cells (approximately 9 versus 0, resp., in the fields shown). Capsids in the cytoplasm of NFV-treated cells were almost exclusively nonenveloped (indicated by arrowheads; approximately 16 versus 23 in panels (c) and (d), resp.).

As shown by Kalu et al. [25] and supported by our preliminary experiments (data not shown), late HSV-1 gene expression in HF cells was not reduced by NFV treatment. We therefore explored the effects of NFV on HSV-1 infected HF cells by transmission electron microscopy. Normal numbers of virus particles appeared to be produced in NFV-treated cells. Similar numbers of capsids were observed in the nucleus of untreated and NFV-treated cells (approximately 39 and 44, resp., Figures 4(a) and 4(b)). However, compared with untreated cells, capsids in NFV-treated cells appeared to be disproportionately retained within the cytoplasm (~14 versus 79, resp.), in which virus particles were more often located outside the plasma membrane (~51 versus 3), suggesting a block in virus maturation or egress. In addition, in contrast to those in the cytoplasm of untreated cells, cytoplasmic capsids in NFV-treated cells were rarely observed to be enveloped (~9 versus 0 in the fields shown; Figures 4(c) and 4(d)). Interestingly, although endoplasmic reticulum (ER) stress, the unfolded protein response (UPR), and autophagy are well known effects of NFV [16, 26–29], neither ER dilation nor the abundance of double-membrane bound vesicles consistent with autophagosomes appeared consistently different between NVF-treated and untreated HSV-1-infected cells.

### 3.5. Glycoprotein Posttranslational Processing and Localization Are Impaired by NFV

**Figure 5:**
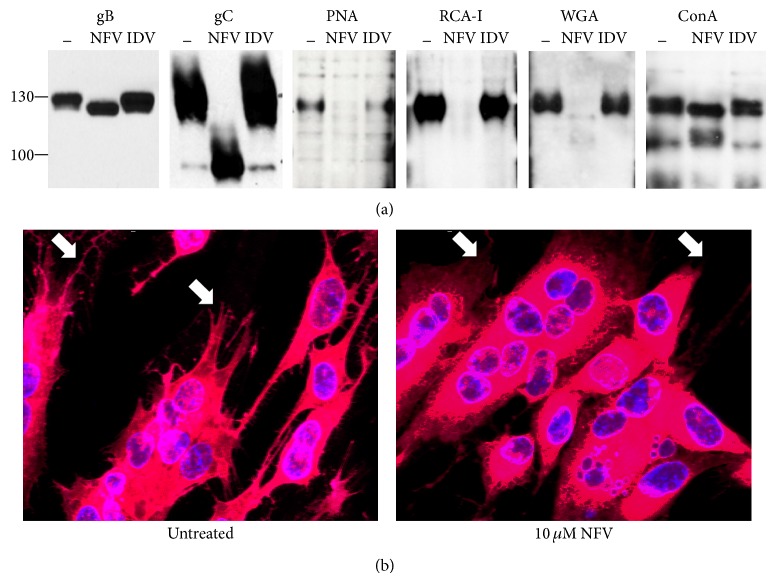
NFV affects the glycosylation and subcellular localization of viral gene products but does not change the level of expression. (a) Western blots of HSV-1 infected HF cells show increased mobility in gB and gC with NFV treatment, compared to cells that were untreated or treated with indinavir (IDV). Eastern blots show that altered staining by lectins (peanut agglutinin, PNA; ricinus communis agglutinin I, RCA-I; wheat germ agglutinin, WGA; and concanavalin A, ConA) demonstrates reduced overall glycosylation in NFV-treated cells. (b) HSV-1 gB localization (red staining) is altered in NFV-treated HF cells, showing decreased delineation of plasma membrane processes (indicated by white arrows) by IFA compared to untreated cells. Nuclei are stained blue.

During evaluation of HSV-1 glycoproteins gB and gC expression by Western blotting, it was apparent that NFV treatment of infected cells resulted in increased electrophoretic mobility (Figure 5(a)) compared to untreated controls or IDV-treated cells. The apparent change in molecular weight of these viral proteins was estimated to be consistent with the reduction in glycosylation [30–32]. To assess the effect of NFV on protein glycosylation in HSV-1-infected cells, Eastern blotting was performed using lectins PNA, RCA-I, WGA, and ConA (Figure 5(a)). Compared to untreated or IDV-treated cells, NFV resulted in a marked reduction in staining by PNA, RCA-I, and WGA indicating decreased addition of galactose, N-acetyl-D-galactosamine, and N-acetyl-D-glucosamine [33, 34]. In contrast, ConA staining was not appreciably reduced, suggesting relatively normal levels of oligomannose-type N-glycans. NFV resulted in a clear alteration in the subcellular localization of HSV-1 gB (Figure 5(b)) by immunofluorescent antibody staining. Compared with control treatments in which viral envelope glycoprotein staining uniformly delineated the plasma membrane of HSV-1-infected HF cells, with NFV treatment staining appeared predominantly perinuclear, suggesting improper trafficking to the cell surface. Consistent with the electron microscopy results, using immunofluorescence, no increase in LC3-II staining, a marker of autophagy, was apparent (data not shown).

## 4. Discussion

NFV is an HIV aspartyl protease inhibitor that, in addition to its antiretroviral activity, has complex effects on numerous human cellular functions, including on Akt signaling, inhibition of cellular proteases, and induction of ER stress, many of which might contribute to its ability to broadly inhibit tumor cell growth as well as replication of several non-HIV viruses [9, 11, 16, 17, 35, 36]. In this study, we specifically explored the mechanism(s) by which NFV might inhibit production of infectious HSV-1* in vitro*. We found that NFV acts on HSV-1 late in virus production, without a detectable effect on late viral gene expression. Furthermore, abundant virus particles were observed within infected cells, though envelopment and release of virus appeared to be substantially diminished. This is consistent with a recently published study by Kalu et al., which also reported that NFV blocked HSV-1 maturation and egress [25].

NFV showed no activity on the VP24 protease, which was expected given that it is a serine protease that lacks structural or functional similarity with the HIV (aspartyl) protease. This was shown using a transfection system to examine enzymatic activity in trans and supports the finding that scaffold protein cleavage appears unaffected in HSV-1-infected cells treated with NFV [25]. No resistance to NFV could be selected under conditions that readily resulted in acyclovir-resistant HSV-1, which is again consistent with findings by Kalu et al. [25]. Though NFV-resistant HSV-1 may well be isolated using other conditions, this finding suggests a relatively high barrier to resistance* in vitro* and suggests a mechanism of action on a host cell function required for virus production, rather than a direct effect on a viral target [37–41]. Indeed, many of the cellular functions affected by NFV have similarly been described to play a role in HSV-1 replication.

NFV inhibits cellular proteases and the proteasome, which leads to accumulation and inefficient removal of misfolded proteins in the ER and Golgi [16, 42, 43]. The finding that NFV resulted in impaired viral protein glycosylation and trafficking is consistent with these processes and again validates the recent findings by Kalu et al. [25]. Of note, based on ConA staining, N-linkage of immature (high mannose) carbohydrates appeared relatively normal [33]. These mannose structures are largely assembled in the cytoplasm, whereas trimming and modification of more complex sugar residues occur in the ER and Golgi. We found that the impairment of viral glycoprotein processing is at least one mechanism by which NFV reduces infectious HSV-1 production. Agents that induce ER stress, such as thapsigargin, similarly interfere with HSV-1 glycoprotein posttranslational processing and production of infectious virus [31]. Numerous studies have reported that tunicamycin, which blocks the synthesis of the N-acetylglucosamine-lipid intermediates, and other inhibitors of protein glycosylation decrease the infectious yield of HSV-1* in vitro* [44–46]. Furthermore, tunicamycin does not affect the level of late viral gene product expression, and normal appearing capsids were noted within the cytoplasm, similar to the effects we observed with NFV. It is unclear, however, whether impaired HSV-1 envelope protein glycosylation would block virus egress based on studies using cell lines deficient in N-acetylglucosaminyl transferase activity, in which virus yield was only mildly reduced [47].

This work has several important limitations. The effects of NFV are highly pleiotropic, and we stress that NFV might affect the production of infectious HSV-1 through multiple mechanisms. In addition, based on what is known about NFV's effects on tumor cells [11, 21], the most relevant mechanism(s) of action may differ with respect to individual HHV, cell type, and drug concentration. By necessity, all of the possible mechanisms by which NFV might affect HSV-1 replication were not evaluated.

Autophagy, a catabolic process that maintains cellular homeostasis under conditions of stress, is a prominent effect of NFV [11, 16]. HSV-1 encodes genes to block autophagy in infected cells, including infected cell protein (ICP) 34.5, which is required for neurovirulence [48]. Increased levels of autophagy can reduce production of infectious HSV-1 similarly to NFV, with relatively normal viral gene expression and formation of viral particles that are retained within cells [49, 50]. We were not able to formally show that NFV increased autophagy in HSV-1 infected cells under the conditions used. NFV treatment appeared to result in retention of cytoplasmic virus particles within single membrane-bound vesicles that could be late autophagosomes as in other reports [49, 50]. However, pathognomonic double-membrane structures were not convincingly observed in greater numbers in NFV-treated versus untreated HSV-1-infected cells nor were we able to show that NFV increased LC3-II staining during HSV-1 infection. Autophagy can be difficult to demonstrate [51] and cannot currently be excluded as a potential contributor to the effect of NFV on HSV-1 or other HHVs [48].

NFV is an orally bioavailable, FDA-approved treatment for HIV infection and is well tolerated during long-term use. Since the advent of more potent and convenient options, NFV is no longer widely prescribed for ART [4]. However, because of its activity against a broad range of tumor cell types, there is intense interest in repositioning NFV as a cancer chemotherapeutic agent, perhaps at higher doses than those for ART [11, 42, 52]. NFV could also be beneficial in the treatment or suppression of infection with HSV and other HHVs, particularly in the setting of resistance to first-line antivirals, in patients with cancer and/or HIV. Additional studies are indicated to further elucidate the mechanism(s) of action of NFV on HHV infections and to evaluate its efficacy in clinical trials.
